# Intraosseous contrast administration for emergency computed tomography: A case-control study

**DOI:** 10.1371/journal.pone.0217629

**Published:** 2019-05-31

**Authors:** Philipp Schindler, Anne Helfen, Moritz Wildgruber, Walter Heindel, Christoph Schülke, Max Masthoff

**Affiliations:** Institute of Clinical Radiology, University Hospital Muenster, Muenster, Germany; Cleveland Clinic, UNITED STATES

## Abstract

**Objective:**

The aim of the study was to evaluate the feasibility of intraosseous (i.o.) contrast media injection (CMI) for emergency computed tomography (CT) of severe trauma and the associated image quality compared to intravenous (i.v.) CMI.

**Materials and methods:**

The authors retrospectively analysed objective (contrast-to-noise ratio (CNR)) and subjective (4-point Likert scale) image quality of CTs after i.o. (n = 4, mean age (y) 57.0±11.0) versus i.v. (n = 20, mean age (y) 58.8±4.4) CMI. All patients underwent a native head CT scan, a cerebral CT angiography (CTA) and CTA of the supra-aortic vasculature as well as a chest and abdominal CT scan in the venous phase; one patient with an i.o. access additionally received a CTA of the lower limbs. Electronic patient records have been reviewed to determine i.o. access related complications.

**Results:**

Both groups were consistent in age, heart rate, scan parameters including the flow rate of the contrast agent, resulting in comparable radiation dose levels. The image noise and CNR had no significant difference between the two groups. Scoring the delineation of the main vessels after i.o. CMI showed no significant difference to the i.v. group. There were no CT or i.o. access related complications observed.

**Conclusion:**

The i.o. access is a safe and suitable alternative for emergency CMI in CT. Using established protocols good to very good image quality can be achieved, comparable to i.v. CMI. We show for the first time, that i.o. CMI is also feasible for CTA imaging of the head and neck region as well as of pelvic and leg vessels.

## Introduction

Severe trauma is a global health problem and the world's leading cause of death for patients under the age of 45 years[1]. Rapid and comprehensive diagnosis of traumatic conditions is critical as patients who receive early computed tomography (CT) after severe trauma reveal significantly increased probability of survival[2]. Therefore, vascular access is of high priority and not only needed for treatment of severely injured patients but also for applying contrast agents enabling for dedicated trauma CT imaging[3,4]. Some patients do not permit rapid peripheral intravenous (i.v.) cannulation (e.g. centralization, obesity). In the case of failed i.v. placement, intraosseous (i.o.) access catheters offer an alternative for drug and volume administration[4,5].

In recent years, the development of purely manual to semi-automatic spring-loaded as well as fully automatic motorized drilling systems has led to facilitated and safe applicability[3,4]. Several professional societies recommend i.o. access as an early alternative for all critically ill patients in the case of failed peripheral venous access[4,6,7]. The most commonly documented complication is extravasation of the applied contrast agent[8,9,10]. Severe complications such as fatty embolisms or osteomyelitis are observed far less frequently and associated to prolonged use of the i.o. access[10,11]. Hence, i.o. access is a well-suited access path in trauma management.

Initial diagnostics of patients with severe trauma commonly involves contrast-enhanced CT assessment. Despite increasing evidence of safe i.o. drug and volume administration, there is scarce information regarding pressure contrast media injection (CMI) in emergency CT as well as regarding the potential need to adjust CT protocols using an i.o. access catheter. So far, knowledge is based on case reports or animal studies[3,12–14].

Therefore, the aim of this pilot study was to evaluate the feasibility of i.o. contrast administration in emergency CT and to assess image quality of i.o. compared to i.v. CMI. Further, we report for the first time a CT angiography (CTA) of the head and neck as well as the pelvis and lower extremity after i.o. CMI.

## Materials and methods

### Study population and CT examination technique

In this retrospective case-control study twenty-four patients who underwent emergency CT as part of trauma management in trauma center were included in the study. Four patients (case group, mean age (y) 57.0±11.0, n = 3 male) had received a tibial i.o. access in case of complicated peripheral venous conditions and consecutive failure of peripheral i.v. cannulation. Twenty consecutive patients (control group, mean age (y) 58.8±4.4, n = 17 male) receiving emergency CT with standard peripheral venous access were matched regarding age and heart rate during the examination (1:5 case-control matching). The study protocol has been approved by the local ethics committee (Ethics committee of the Medical Chamber of Westfalen-Lippe and the University of Muenster, Muenster, Germany; ID: 2019-040-f-S) and all patients provided written informed consent.

Technical placement of the i.o. access often takes place at the scene of the accident or on arrival at the emergency room while in our trauma center it is a task of the anesthetists [11]. Briefly, after identification of the anatomical landmarks the puncture site is disinfected and infiltrated with local anesthesia. Subsequently, the i.o. needle is inserted to the bone marrow usually using a (semi-)automatic device. Consecutively aspiration of bone marrow for verification of cannula position and injection of a test fluid bolus (e.g., 10 cc sodium chloride) should be performed. Finally, the intraosseous access is secured and the infusion line or contrast media power injector can be attached similar to a standard peripheral venous access.

All scans were performed with single source CT (SOMATOM Definition AS, Siemens Healthcare; 64 x 0.6mm x 2 alternating focal spot slice acquisition) according to the institutional trauma protocol and patient-based tube current modulation (CareDose4D, Siemens Healthcare).

Both groups (i.o. vs. iv) were examined with identical routine trauma CT protocols and the same amount and flow rate of iodinated non-ionic contrast medium (ULTRAVIST, Bayer Healthcare) delivered by a power injector. Our trauma protocol in case of an i.o. access additionally includes a short low-dose CT scan over the i.o. device prior to the main examination to confirm correct placement. Main examination starts with a native head CT scan followed by a cerebral CTA and CTA of the supra-aortic vasculature (80cc contrast medium, 4cc/s flow rate) while the patients’ arms are lowered. Then, patients’ arms are elevated if possible, followed by a chest and abdominal scan in an early (delay 65s) venous phase after second contrast administration (80cc, 3cc/s). One patient received a CTA of the vasculature of the lower limbs after CT scan of the head and neck. After second contrast administration (test bolus 20cc, CTA with 40cc at 5cc/s and 80cc at 3cc/s) for the CTA of the lower limbs the chest and abdominal scan in an early venous phase was performed without any further contrast administration.

From the raw data set, images with 0.6mm slice thickness were reconstructed for multiplanar reformation. Subsequently, the image data sets were transferred to the PACS (CENTRICITY, GE Healthcare) for diagnosis and further evaluation. Radiation dose parameters were reported as dose-length product (DLP) and volume computed tomography dose index (CTDIvol.).

Any CT or i.o. access related complications during the scan (in particular extravasation) have been documented and electronic patient records have been reviewed to find out complications in the further inpatient course (e.g. fatty embolisms or osteomyelitis).

### Objective image quality

In order to evaluate objective image quality criteria, mean density ± standard deviation and contrast-to-noise ratio (CNR) were measured for both injection pathways according to Feuchtner et al.[15]. Measurements were performed on axial source images by an emergency radiologist at the internal carotid artery, ascending aorta and abdominal aorta. The absolute attenuation in a region of interest (ROI) was expressed as Hounsfield Units (HU) and image noise was calculated as the standard deviation of the mean density (SD of HU).

### Subjective image quality

The subjective image quality of i.o. and i.v. CMI was independently analysed by 3 radiologists (MM, CS, PS) regarding image noise, delineation of relevant vascular structures and their course to the periphery and the overall subjective image quality each using a 4-point scale.

The subjective image noise was evaluated dedicated to the ROI:

1. minimal image noise
2. slight image noise
3. strong image noise
4. extremely strong image noise

The presence of contrast media was evaluated dedicated to the delineation of relevant vascular structures and their course to the periphery on a 4-point scale:

Head/neck (middle cerebral artery), definable to

1. M3-segmental level
2. M2-segmental level
3. M1-segmental level
4. not sufficiently definable

Chest (aorta), definable to

1. extrathoracic supra-aortic branches
2. intrathoracic supra-aortic branches
3. supra-aortic branches cannot be clearly distinguished
4. not sufficiently definable

Abdomen (coeliac trunk/superior mesenteric artery), definable to

1. third bifurcation
2. second bifurcation
3. first bifurcation
4. not sufficiently definable

Finally, we scored the overall image quality on a further 4-point scale:

1. high image quality without relevant limitation (very good)
2. good image quality with moderate image noise (good)
3. limited diagnostic image quality due to strong noise or artefacts (limited)
4. non-diagnostic (insufficient)

Artefacts of medical devices and other overlying material occurred in both groups and were not further evaluated.

### Statistics

Statistical analysis was performed using Prism 8 (GraphPad Software Inc). All data are presented as mean ± standard deviation. The student t-test was used for quantitative data. The Mann-Whitney U-test was used for non-normally distributed, qualitative data. Two-sided p-values < .05 were considered to be statistically significant.

## Results

All examinations were performed without any CT or i.o. access related complications during the scan or in the further inpatient course. Details on patients' characteristics are shown in **Table 1**. There was no significant difference in age and heart rate between the two groups during the CT scan. Using identical examination protocols as well as contrast agent volumes and flow rates as described above comparable dose levels were observed.

**Table 1:** Patient characteristics and radiation dose parameters.

	i.o., n = 4, mean ± SD	i.v., n = 20, mean ± SD	p-Value
Age (y)	57.0 ± 11.0	58.8 ± 4.4	0.872
Heart rate (1/min)	90.5 ± 1.2	79.4 ± 3.3	0.152
CTDI vol. head/neck (mGy)	110.8 ± 7.1	107.8 ± 2.6	0.650
CTDI vol. chest/abdomen (mGy)	13.0 ± 2.7	10.8 ± 0.5	0.181
DLP head/neck (mGy*cm)	2365.0 ± 159.4	2426.0 ± 85.9	0.770
DLP chest/abdomen (mGy*cm)	865.0 ± 183.9	811.5 ± 40.1	0.665

Abbreviations: SD, standard deviation; i.o., intraosseous; i.v., intravenous; CTDI vol., volume computed tomography dose index; DLP, dose length product.

### Objective image quality

To further explore the potential use of i.o. access for CMI in emergency CT we analysed objective image quality parameters and compared them with CT scans after i.v. CMI (**Table 2**). There was no statistical difference in absolute CT-attenuation (mean HU ± SD) between both groups for head and neck (i.o. 303.1±184.7 vs. i.v. 359.4±105.4, p = 0.398), chest (i.o. 200.0±62.4 vs. i.v. 197.7±28.5, p = 0.911) or abdominal (i.o. 201.5±69.0 vs. i.v. 183.8±32.3, p = 0.454) imaging.

**Table 2:** Objective image quality parameters.

	i.o., n = 4, mean ± SD	i.v., n = 20, mean ± SD	p-value
**Absolute CT-attenuation (HU)**			
Head/neck	303.1 ± 184.7	359.4 ± 105.4	0.398
Chest	200.0 ± 62.4	197.7 ± 28.5	0.911
Abdomen	201.5 ± 69.0	183.8 ± 32.3	0.454
**Image noise** **(SD of HU)**			
Head/neck	20.0 ± 11.5	30.5 ± 25.0	0.421
Chest	13.7 ± 1.4	15.3 ± 3.0	0.364
Abdomen	21.3 ± 3.8	22.0 ± 4.2	0.807
**CNR** **(ratio HU/noise)**			
Head/neck	28.7 ± 19.1	26.7 ± 20.1	0.860
Chest	19.3 ± 4.0	17.8 ± 3.3	0.494
Abdomen	13.3 ± 5.5	12.5 ± 2.8	0.687

Abbreviations: SD, standard deviation; i.o., intraosseous; i.v., intravenous; HU, Hounsfield Units; CNR, contrast-to-noise ratio

As shown in **Table 2,** analysis of image noise values (mean SD of HU) after i.o and i.v. CMI revealed again no statistical differences between both groups for head and neck (i.o. 20.0±11.5 vs. i.v. 30.5±25.0, p = 0.421), chest (i.o. 13.7±1.4 vs. i.v. 15.3±3.0, p = 0.364) or abdominal (i.o. 21.3±3.8 vs. i.v. 22.0±4.2, p = 0.807).

Finally, CNR (mean ratio HU/noise) demonstrated no statistically significant differences between both groups for head and neck (i.o. 28.7±19.1 vs. i.v. 26.7±20.1, p = 0.860), chest (i.o. 19.3±4.0 vs. i.v. 17.8±3.3, p = 0.494) or abdominal (i.o. 13.3±5.5 vs. i.v. 12.5±2.8, p = 0.687).

### Subjective image quality

In both groups all data sets were evaluable. All 3 raters analysed a total of 30 (i.o.) vs. 180 (i.v.) image sequences. In the i.o. group the majority showed only a minimal subjective image noise (n = 13, 43.3%) or slight image noise (n = 14, 46.7%). Only 10% (n = 3) showed strong image noise. In the i.v. group the majority also revealed only a minimal subjective image noise (n = 58, 32.2%) or slight image noise (n = 111, 61.7%), at 6.1% (n = 11) the image noise was subjectively strong.

There was no statistical difference in the delineation of all evaluated vessels between the i.o. and the i.v. group (median score 1.0 vs. 1.0; mean 1.2±0.4 vs. 1.1±0.3; p = 0.405).

Again, there was no statistical difference in the overall image quality score between the i.o. and the i.v. group (median score 2.0 vs. 1.5; mean 1.75±0.45 vs. 1.52±0.54; p = 0.196) as well as in the subgroup analysis.

### CT findings

**Fig 1:**
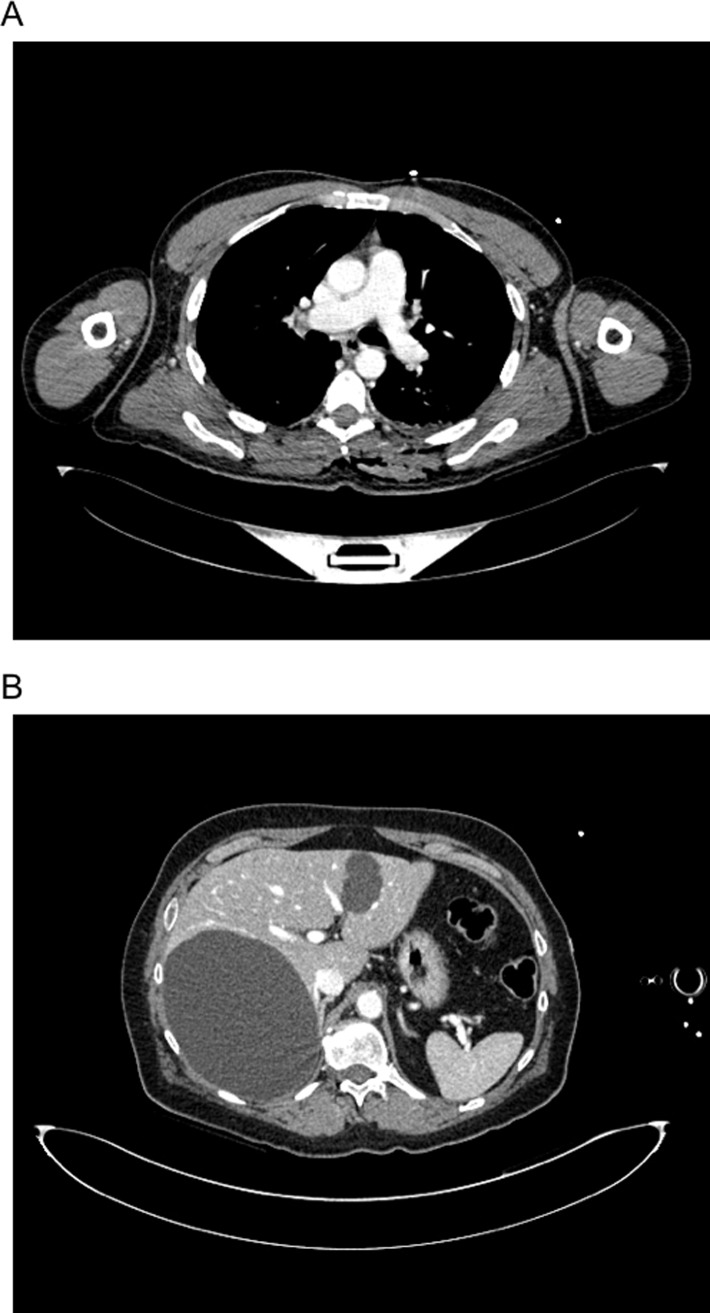
Feasibility of contrast enhanced CT via i.o. access with very good enhancement of the major thoracic vessels (A) and parenchymatous upper abdominal organs (B).

**Fig 2:**
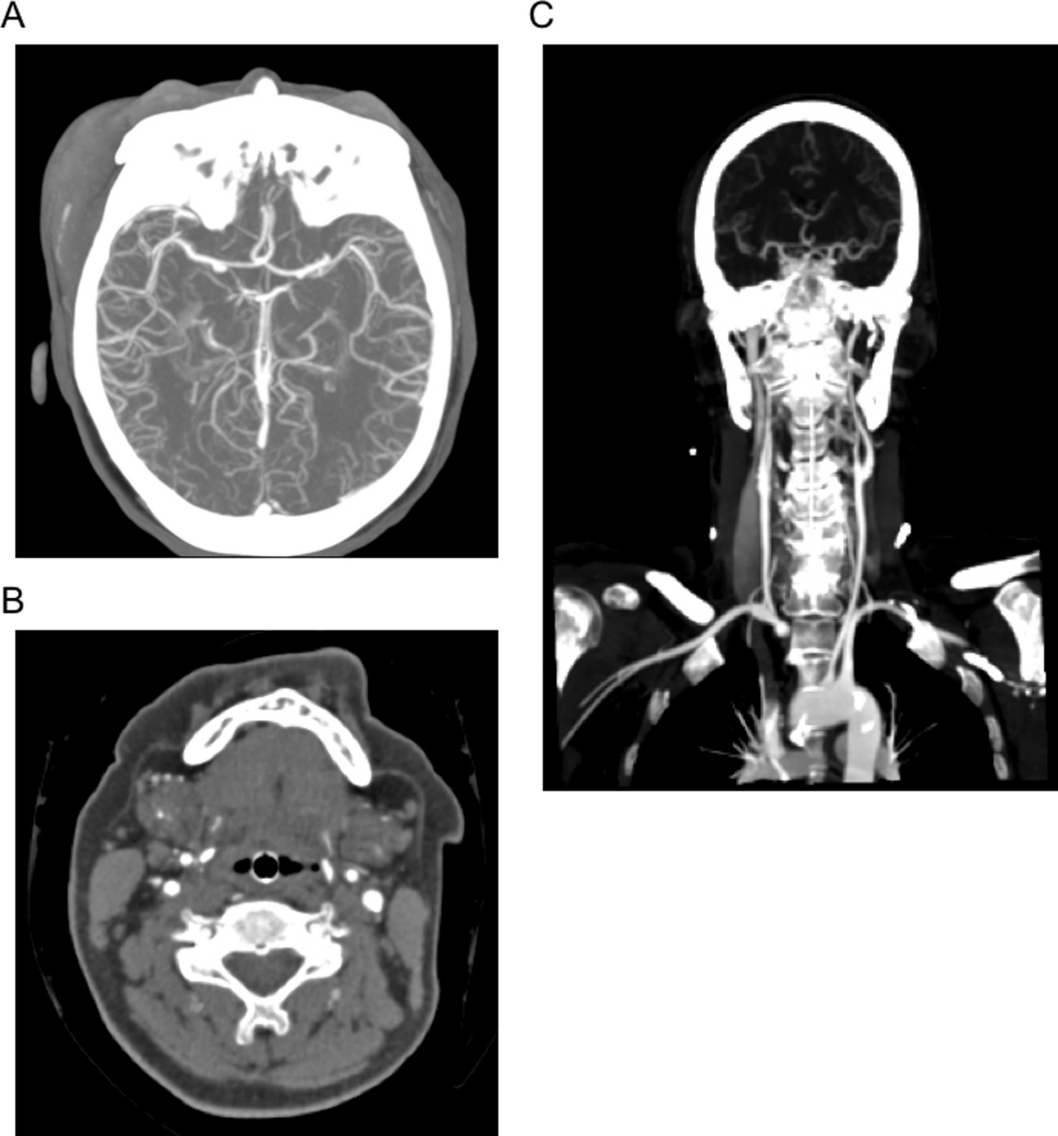
CTA of the head and neck via i.o. access. (A) Maximum intensity projection (MIP) axial reformatted image of the cerebral arteries with excellent enhancement and delineation up to distal segment levels. (B) Very good image quality with excellent enhancement of internal and external carotid arteries. (C) Maximum intensity projection (MIP) coronal reformatted image of the supraaortic branches.

**Fig 3:**
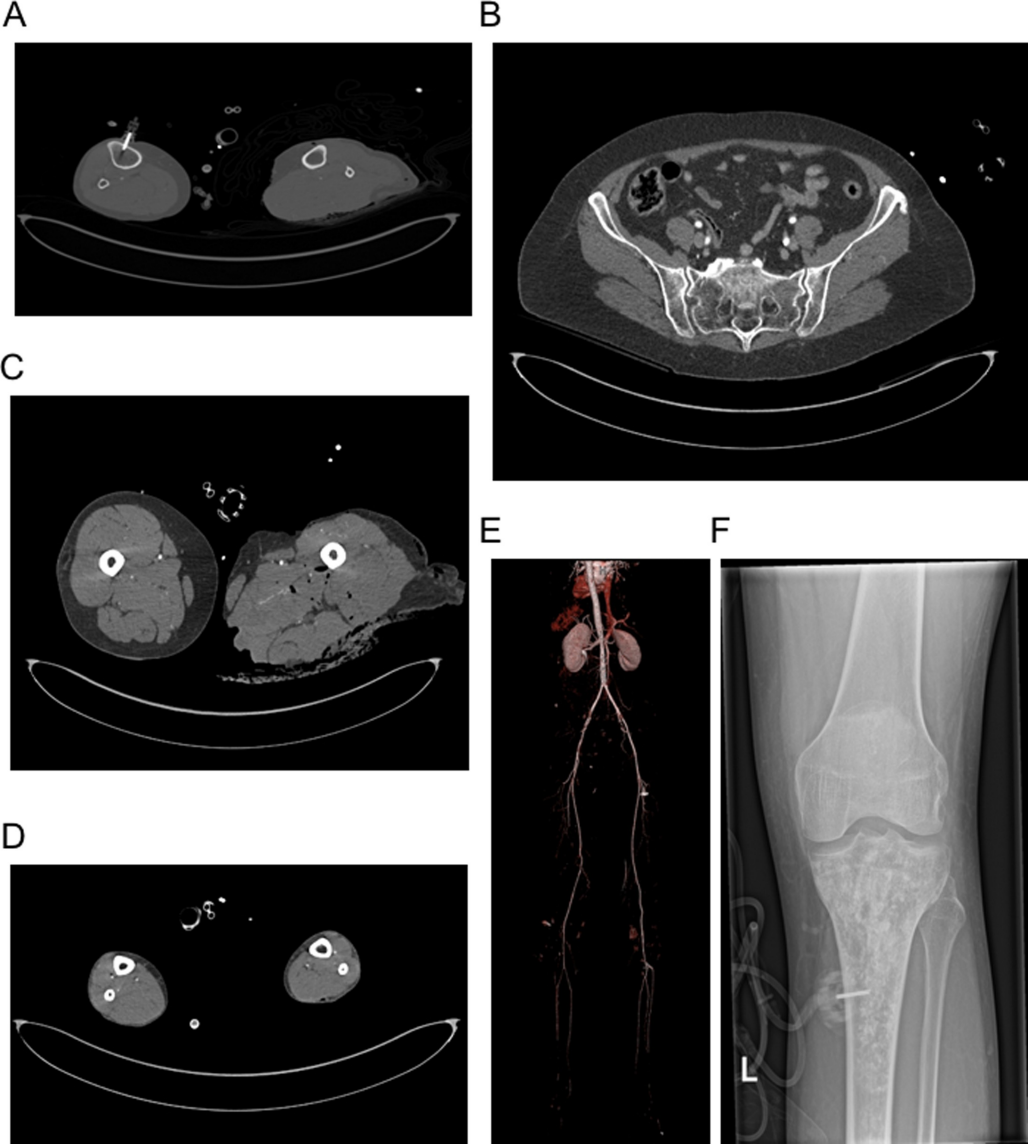
CTA of the pelvic and leg vessels via i.o. access. (A) Short CT scan over the i.o. device prior to the main examination to confirm placement within the intramedullary cavity. Very good image quality with sufficient enhancement of the internal and external iliac arteries (B) as well as in the course of femoral arteries (C) and tri-vessel enhancement of the tibiofibular tract (D). (E) Volume-rendering reconstruction of the abdominal aorta and pelvic and leg vessels. (F) X-ray image of the knee of another patient (as A to E) of the i.o. group with correct placement of the i.o. device and contrast enhanced intramedullary cavity after CT scan.

Since a limited number of studies have examined the feasibility of i.o. CMI in a chest and abdominal CT scan, we were able to perform contrast enhanced CTs via i.o. access with very good to good image quality as described above and sufficient enhancement of the major vessels, mediastinum and abdominal parenchymal organs, as shown in Fig 1. We present the first report of a CTA via i.o. CMI of the head and neck region as well as of pelvic and leg vessels. Fig 2 shows the very good contrast enhancement of the supra-aortic and cerebral arteries, exemplified by the delineation of the middle cerebral artery down to the M3 segmental level. Fig 3A shows short confirmation scan of correct intramedullary placement of the i.o. access. Fig 3B–3F shows exemplary images with very good contrast of the pelvic and leg arteries with tri-vessel contrasting of the tibiofibular tract.

## Discussion

We explored the feasibility of i.o. CMI in emergency CT in trauma management and compared the image quality to i.v. contrast enhanced CT.

We demonstrated that i.o. CMI could be performed using established CT protocols with identical contrast medium amount and flow rate comparable to i.v. CMI. Here, no complications were observed. In all examinations we obtained a good to very good image quality of the chest and abdomen comparable and without significant difference to i.v. CMI. In addition, for the first time we were able to perform a CTA of the head and neck region as well as of pelvic and leg vessels. This turns out the i.o. access is also suitable for complex CT examinations with high flow rates up to 5cc/s.

Using a swine model, Cohen et al. described the concept of CT with i.o. CMI[3]. Here, as a crossover study each swine (n = 8) underwent i.o. and i.v. contrast administrated CT, while all images acquired by the i.o. route were rated as adequately enhanced by two radiologists. Ahrens et al. and Budach et al. each described in a case report (each n = 1) the feasibility of a CTA of the pulmonary arteries via i.o. access with 5cc/s or CTA of the chest and abdomen with 4cc/s with good quality and no complications[12,13]. Winkler et al. also demonstrated the feasibility of thoracic CTA via i.o. access in a larger number of cases (n = 17) as a safe and effective route of CMI up to 4cc/s[13]. As the main focus of their study was the thoracic aorta they also determined the absolute attenuation of the aorta and CNR to objectify the image quality, but there was no comparison to the i.v. access route.

Two paediatric case reports (each n = 1) have been published using manual injection for trauma CT imaging in case of an i.o. access[16,17]. We were able to perform the study with power injection. Using a power injector, the radiologist does not have to stand near by the CT scanner and risk of radiation exposure is reduced. In addition, high flow rates (up to 5cc/s) and a volume of up to 100cc are necessary for the CTA of the head and neck region as well as pelvic and leg vessels. Using power injectors these flow rates and volumes can be reached in a much more reproducible manner.

The study focused on the feasibility of CT examination via i.o. access while the technical placement of the i.o. access was not the focus of the study. Some authors discuss a short low-dose CT scan over the i.o. device prior to the main examination to confirm correct placement within the intramedullary cavity[4]. As this is a simple, elegant method to diagnose misplacements and to avoid side effects after CMI, we do also recommend to conduct a low-dose CT as part of routine protocol in patients with i.o. access prior to CMI.

The current study was performed as a feasibility study and is limited by a low patient number demanding for further studies. However, the results demonstrate that i.o. access provides a fast and safe alternative for emergency CMI in CT with good imaging quality comparable to standard i.v. CMI. Moreover, first description of CTA of the head and neck region as well as pelvic and leg vessels support i.o. access as suitable alternative for emergency vascular imaging. In particular, the simple and secure handling and use of standardized routine protocols allow implementation into the clinical routine without reducing image quality.
